# Retraction: Overexpression of NR4A1 is associated with tumor recurrence and poor survival in non-small-cell lung carcinoma

**DOI:** 10.18632/oncotarget.28396

**Published:** 2023-03-24

**Authors:** Bing Zhu, Jian-Ru Yang, Yang Jia, Pei Zhang, Lin Shen, Xiao-Long Li, Jing Li, Bing Wang

**Affiliations:** ^1^Department of Cardiothoracic Surgery, The Second Affiliated Hospital of Chongqing Medical University, Chongqing 400010, P.R. China; ^2^Central Laboratory, Handan Infectious Diseases Hospital, Handan 056002, P.R. China; ^3^Department of General Practice, The Second Hospital of Hebei Medical University, Shijiazhuang 050000, P.R. China; ^*^These authors have contributed equally to this work

**This article has been retracted:** Oncotarget is retracting this article at the request of the Scientific Integrity office. Similarities were found between images in this article and in other articles:

1. BMC Cancer (2017), https://doi.org/10.1186/s12885-017-3132-9

2. Disease Markers (2017), https://doi.org/10.1155/2017/6136401

Reader concerns were initially reported in a Pubpeer thread (https://pubpeer.com/publications/1D3EFEEE1E55BFC3B47D193AEFECE6).

Image analysis performed by the Oncotarget Scientific Integrity Office confirmed the findings of fraudulent image duplication. All authors have agreed to this retraction.

Original article: Oncotarget. 2017; 8:113977–113986. 113977-113986. https://doi.org/10.18632/oncotarget.23048

